# Development and application of triage and medical evacuation system for casualties at sea

**DOI:** 10.1186/2054-9369-1-12

**Published:** 2014-06-01

**Authors:** Tai Xie, Xiao-Rong Liu, Guo-Liang Chen, Liang Qi, Zhi-Yin Xu, Xu-Dong Liu

**Affiliations:** Department of Health Service, Second Military Medical University, 800 Xiangyin Road, Shanghai, 200433 China

**Keywords:** Triage, Medical evacuation, Equipment, Mass casualties, Navy

## Abstract

Traditional triage could not meet the needs of battlefield casualties’ care in modern warfare. This paper designs of triage and medical evacuation system for casualties at sea that can quickly address mass-casualty triage, and store and transmit medical information during battlefield treatment and medical evacuation. This system consists of a high-capacity medical information card, a simulated patient generator, a triage classifier and a multifunctional airbag triage vest.

## Introduction

The 21st century has seen a tendency for human to move towards the ocean. Rights to and interests in the ocean are deeply related to the survival and development of countries. It is the mission and duty of a country’s navy to protect the country’s marine rights and interests. In recent years, as the fights for the ocean have become much more fierce and complicated, China has strengthened efforts in protecting marine interests. Enhancing the overall effectiveness of naval battles and safeguarding marine rights and interests by continuously improving the health service support capability in navy have become a significant focus. Because the marine battlefield is spacious and unsheltered and the shipboard is space-constrained with concentrated duty posts and vulnerable sailors, various types of wounded troops and different conditions will be generated in a short period of time once a marine battle occurs. Because of limitations in rescue time and capabilities, contradictions between immediate and potential rescues emerge, the number of casualties requiring emergency care can exceed the resources available. To improve the situation, the sick and wounded must be rapidly and accurately triaged into one of the following categories: priority 1, immediate; priority 2, urgent; priority 3, delayed or hold; priority 4, expectant. This triage method allows medical personnel to ration limited medical resources to take medical care in a way that handle the casualties as many as possible. Traditionally, triage is performed by the triage surgeon by checking triage tags, inquiring about the injury, observing a patient’s physiological status and writing triage tags. Furthermore the efficacy of this triage system relies on the surgeon’s personal medical ability and experience, which may not always be accurate [1]. In addition, the paper triage tags are hard to fill out in battlefield. Traditional paper triage tags and instruments cannot meet the needs of battlefield medical rescue and evacuation in a timely manner in the information age.

Based on practical battlefield needs, we developed triage and medical evacuation system for casualties at sea that successfully achieved the quick triage of mass casualties and the storage and transmission of information in emergency treatment and medical evacuation, which can significantly improve the efficiency and enhance capability for battlefield medical rescue.

### Design and methods

The triage and medical evacuation system for casualties at sea consists of a high-capacity medical information card, a simulated patient generator, a triage classifier and a multifunctional airbag triage vest. The high-capacity medical information card acts as a carrier to record and transmit information for mass casualties; the classifying surgeon reads the information card with the classifier to retrieve the integrated casualty information. The simulated patient generator can be used to generate simulated mass casualties from the database that can generate a large number of casualties with different kinds of trauma for simulated training, which improves the medical providers’ treatment capacity. Once the simulated information is received by the classifier, the triage service module will generate a trauma score, perform triage and transmit the data to the database. Comprehensive information about the casualties in the database can be read by the doctor workstation and the nurse workstation and then medical manipulations, such as performing surgery, issuing medical orders, writing triage tags and conducting examinations, can be conducted. The resulting data will then be recorded and analyzed. The system can be used to automate and standardize data and processes, enable faster and more accurate information flow and improve the surgeons’ performance. The airbag multifunctional triage vest can effectively protect the safety of the surgeons, who can use the medical equipment to perform the primary care. It is very important to save time for effective treatment during the “platinum 10 minutes” and the “golden time” emergency period.

### Components and implementation

#### High-capacity medical information card

**Figure 1:**
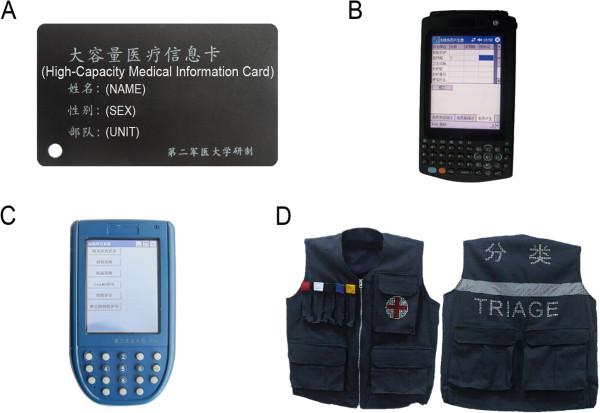
**Four components of triage and medical evacuation system for casualties at sea. (A)** The high-capacity medical information card, which has an RFID chip inside and a PVC plastic shell outside, can identify wounds, track casualties through triage process and transmit information to institutions at all levels. **(B)** The simulated patient generator can provide specific types of casualties for the simulation training of medical personnel, which increases the training intensity and improves the rescue abilities of medical personnel. **(C)** The Triage classifier can capture the casualties’ information, generate the trauma index and conduct the casualty triage, which can guide medical personnel in providing first aid to the wounded and performing medical evacuations. **(D)** The multifunctional airbag triage vest is composed of an airbag device, a multipocket design and fluorescent strips, and it is capable of carrying colored markers and other medical instruments. Red tags indicate hemorrhage, White tags indicate fracture, Black tags indicate infectious disease, Blue tags indicate radiation disease and Yellow tags indicate biological agents.

The development of the medical information card has inverted the traditional battlefield paper medical document, which is used to identify wounds and track the casualties through the triage process. Since World WarI, many countries have attached great importance to medical evacuation files and provided their own triage tags and battlefield record formats [2, 3]. Some countries use a nationally standardized triage tag, while other countries use commercially available triage tags. However, paper-made records can be easily damaged, which is not conducive to transmitting information to institutions at all levels [4, 5]. The medical information card utilizes radio frequency identification (RFID) technology; an RFID chip that follows ISO14443 standards is inside and it is protected by a PVC plastic shell which is high- and low-temperature resistant, waterproof and corrosion-proof (Figure 1A).

Using modern information technology, the high-capacity medical information card combines RFID technology with mobile storage technology. The card not only features RFID technology’s advantages of low cost, contactless reading and good performance, but also has the removable storage device characteristics of large storage capacity and high reading/writing speed. With integrated circuit technology, the card has been extremely compressed to be just smaller than an ordinary ID card and it can be worn around the neck of the casualty. With chip technology, the card has a maximum storage capacity of up to 6 GB. A certain amount of information can be stored and the storage space is sufficient to ensure the adequate integrity and consistency of the information store based on practical needs and reasonable cost. In addition, the medical information card requires the use of special tools, including a supporting handheld classifier, a PC add-in card reader (with USB interface) to read and write the information on the card. The card also requires dedicated software to retrieve the information and ensure the information’s security, even if the card is lost. With all this technology, the high-capacity medical information card is easy to carry and provide good storage ability, confidentiality and other characteristics.

#### Simulated patient generator

The simulated patient generator developed can prerecord the wounded models and cases by the simulating a wounded management station before the training and compiling a casualty information database [6]. The simulated patient generator features a master control CPU module, a Wi-Fi wireless transmission module and an RFID reading and writing module. The RFID reading and writing device is compatible with 3 types of standards [7]: ISO14443A/B and IS015693 with a 5 cm reading distance and an RF field work frequency (Fc) of 13.56 MHz. The master control CPU has an ARM processor architecture with features such as small volume, low power consumption, low cost and high performance and the ARM processor has a 32-bit reduced instruction set (RISC) processor architecture, which has been widely used in many embedded system designs (Figure 1B).

The casualty information database records the basic information of the wounded. During the simulation training process, the casualties’ information can be sent from the database to the triage group according to specified methods. The database can provide specific types of casualties for the medical simulation training and it can show a variety of trauma types to medical personnel at the same time, which increases the intensity of training and improves the medical personnel’s ability to rescue and treat the patients. According to experience from previous exercises, when medical provider meet critical patients, they often cannot accurately and properly judge or treat the traumatic casualties. Based on traditional traumatic and disease conditions, the patient generator uses a database that contains a variety of traumatic and disease conditions, which has improved medical personnel’s mastery of rescuing and treating casualties. After the casualties are classified by the medical personnel who are responsible for the triage work, their information is sent to the workstations automatically. The doctor workstation and the nurse workstation for every rescuing department are compatible with the Hospital Information System (HIS).

#### Triage classifier

The triage classifier can easily and quickly capture the casualties’ information, generate the trauma index and conduct the casualty triage, which can guide the battlefield surgeon in providing first aid and performing medical evacuations. The classifier transmits real-time casualty information to the servers of medical institutions through wireless LAN and can automatically analyze the information. These data are then transmitted to the base hospital that will receive the patients via telemedicine, which can achieve timely communication with the echelon hospital. The system is compatible with the COMPASS Navigation Satellite System for real-time communications and is integrated with HIS [8]; it supports information reading, storing and sharing and offers information retrieve functions (Figure 1C).

The triage classifier can help the military triage surgeon easily and quickly read the high-capacity medical information card, get complete medical information and fill in the triage tags via a touch screen. The intelligent triage software installed in the classifier can generate a trauma index and provide scientific references for triage capability. Six types of current international trauma score methods have been integrated into the intelligent triage software, including the Simplified Trauma Score (STS), the Trauma Index (TI), the Pre-Hospital Index (PHI), CRAMS, the Trauma Score (TS) and the Revised Trauma Score (RTS) [9, 10]. The Simplified Trauma Score is mainly used in the battlefield trauma assessment and scoring and the other methods are mainly used in military operations other than war, such as military medical support during disaster relief [11]. Introducing trauma scoring methods into mass causalities triage, can increase the accuracy of and scientific support for trauma assessment and can promote the application of information technology to treatment on the battlefield, thus improving its efficiency. Teaching applications of the triage classifier could improve medical personnel’s awareness and understanding of information technology equipment development, which is of great importance to battlefield medical treatment.

#### Multifunctional airbag triage vest

In light of the particularity and complexity of the marine environment and the characteristics of naval medical support, we have designed and developed a multifunctional airbag triage vest dedicated to marine casualty triage. The multifunctional airbag triage vest is wholly composed of an airbag device, a multipocket design and fluorescent strips. The airbag device of the multifunctional airbag triage vest is made up of mutually independent airbags, which are up-down extended long strips arranged from left to right [12]. The airbag is connected to a check valve via a pipe. Other components include a safety valve, an automatic valve, a leash, an inflatable nozzle, a buckle, rings and a carbon dioxide cylinder. The airbag device is closely joined with the overall structure of the triage vest in an appropriate manner (Figure 1D).

Longitudinally oriented strip pockets are located outside the front of multifunctional airbag triage vest on the right front of the chest. The square paste area is on top of the pockets. All five of the strip pockets, which are used to place wound markers, correspond to different colors. The paste areas outside of the pockets can be pasted with different colored markers according to the patient’s condition. The colored markers are 15 × 3.5 cm cloth or plastic strips of a specific color. Wound markers representing different special kinds of injuries can attract the attention of providers at different levels to recognize the need for prioritized treatment, evacuation or other protective measures for the wounded.

The surgeons who wear the multifunctional airbag triage vest could use personal digital assistant to quickly assess injuries and provide effective and reasonable triage. Furthermore, medical treatment and evacuation can be prioritized according to the injury severity. The multipocket layout design of the multifunctional airbag triage vest is capable of carrying wound markers, sorting markers, a sphygmomanometer, a stethoscope, a one-handed tourniquet, hemostatic bandages, roll splints, mouth gags, tongue forceps, dental pads, triangle bandages, gauze and other medical instruments. This design allows care providers to do primary treatment to casualties with specific injuries, which could save time for medical evacuation and further treatment. At the same time, we have specially designed the water-saving device to include mutually independent airbags, which could protect the medical staff’s safety by effectively lowering the drowning hazard for marine medical rescue staff in cases of accidental falls into water.

### Application and discussion

The triage and medical evacuation system for casualties at sea has played a significant role in guiding the troops’ military exercises and the hospital exercises. In the support exercises of military operations other than war organized by the 411th Hospital and 413rd Hospital and the “Harmonious Mission 2010” and the “Harmonious Mission 2011” held on the hospital ship “Peace Ark”, the application of the triage and medical evacuation system improved the overall treatment efficiency and promoted the progress of the security information technology reform, which has been reported by the China Central Television (CCTV) in 2009. The system was proven effective and practical by the “Marine Health Service Exercises 2009” organized by the Second Military Medical University and the Donghai Fleet of PLA. In that exercise more than 300 casualties were rescued and treated successfully using the equipment, proving that the equipment and the associated information system achieve their general goal and tactical index and were of great help in advancing the modernization and scientific progress of military health service training. The triage and medical evacuation system for casualties at sea takes full advantage of modern information technology to create a convenient classified service operation mode for information about the wounded. It also addresses the traditional triage methods’ accuracy deficits, improving the accuracy of and scientific support for injury evaluation and promoting the application of information technology to battlefield casualty assessment and treatment. The efficiency of marine battlefield treatment has improved as a result.

## Abbreviations

HIS: Hospital information system
PHI: Pre-hospital index
RFID: Radio frequency identification
RTS: Revised trauma score
STS: Simplified trauma score
TI: Trauma index
TS: Trauma score.
